# Supplementary material for: Polar Bloch points in strained ferroelectric films
Source: Nat Commun. 2024 May 10;15:3949. doi: 10.1038/s41467-024-48216-1 (PMC11087520; doi:10.1038/s41467-024-48216-1)
Supplement: Supplementary file 1 — Supplementary Information [file 41467_2024_48216_MOESM1_ESM.pdf]

Supplementary Materials for:

Polar Bloch points in strained ferroelectric films

Yu-Jia Wang[1]†, Yan-Peng Feng[2,3]†, Yun-Long Tang[1], Yin-Lian Zhu[2,3,4], Yi Cao[1,5], Min-Jie Zou[2,3], Wan-Rong Geng[2,3], and Xiu-Liang Ma[2,3,6,7]*

[1]Shenyang National Laboratory for Materials Science, Institute of Metal Research, Chinese Academy of Sciences, Wenhua Road 72, 110016 Shenyang, China.

[2]Bay Area Center for Electron Microscopy, Songshan Lake Materials Laboratory, Dongguan 523808, Guangdong, China.

[3]Quantum Science Center of Guangdong-HongKong-Macau Greater Bay Area, Shenzhen, China

[4]School of Materials Science and Engineering, Hunan University of Science and Technology, Xiangtan 411201, China

[5]School of Materials Science and Engineering, University of Science and Technology of China, Wenhua Road 72, Shenyang 110016, China.

[6]Institute of Physics, Chinese Academy of Sciences, Beijing 100190, China.

[7]State Key Lab of Advanced Processing and Recycling on Non-ferrous Metals, Lanzhou University of Technology, 730050 Lanzhou, China

†Authors contributed equally in this work.
*Correspondence should be addressed to Xiu-Liang Ma (xlma@iphy.ac.cn)

This file contains:

Supplementary Note 1
Supplementary Figures 1-15
Supplementary Table 1

Supplementary Note 1. The effect of the top electrode in the phase-field model.

When we solve the mechanical equilibrium equation $\sigma_{ij,j} = 0$, the whole system, including the ferroelectric film and the top and bottom electrodes, is considered. The elastic constants of two electrodes are adopted to be the same as those of PTO for the sake of simplicity. The total elastic energy density can be written as a piecewise function:

$$f_{elas} = \begin{cases} \frac{1}{2}C_{ijkl}\varepsilon_{ij}(x,y,z)\varepsilon_{kl}(x,y,z), & -h_{be} \le z < 0 \\ \frac{1}{2}C_{ijkl}\left[\varepsilon_{ij}(x,y,z) - \varepsilon^0_{ij}(x,y,z)\right]\left[\varepsilon_{kl}(x,y,z) - \varepsilon^0_{kl}(x,y,z)\right], & 0 \le z \le h_f \\ \frac{1}{2}C_{ijkl}\varepsilon_{ij}(x,y,z)\varepsilon_{kl}(x,y,z), & h_f < z \le h_f + h_{te} \end{cases} \quad (1)$$

where h_f, h_{te} and h_{be} are the thicknesses of the ferroelectric film and the top and bottom electrodes, respectively. In our model, h_f and h_{be} are constant values and only h_{te} varies. As a result, f_{elas} is a function of h_{te}. After solving the mechanical equilibrium equation $\sigma_{ij,j} = 0$, one can obtain the stress tensor σ_{ij}, which is also a function of h_{te}. The mechanical driving force $-\frac{\partial f_{elas}}{\partial P_i}$ in the ferroelectric film can be deduced as:

$$-\frac{\partial f_{elas}}{\partial P_i} = \frac{1}{2}C_{klmn}\left[\frac{\partial \varepsilon^0_{kl}}{\partial P_i}(\varepsilon_{mn} - \varepsilon^0_{mn}) + (\varepsilon_{kl} - \varepsilon^0_{kl})\frac{\partial \varepsilon^0_{mn}}{\partial P_i}\right] = C_{klmn}\frac{\partial \varepsilon^0_{kl}}{\partial P_i}(\varepsilon_{mn} - \varepsilon^0_{mn}) = \sigma_{kl}\frac{\partial \varepsilon^0_{kl}}{\partial P_i}$$
$$= \sigma_{kl}(Q_{klit}P_t + Q_{klsi}P_s) = 2\sigma_{kl}Q_{klit}P_t = 2Q_{ijkl}\sigma_{kl}P_j \quad (2)$$

During the deduction, we substituted $f_{elas} = \frac{1}{2}C_{klmn}(\varepsilon_{kl} - \varepsilon^0_{kl})(\varepsilon_{mn} - \varepsilon^0_{mn})$ and $\varepsilon^0_{kl} = Q_{klst}P_sP_t$. As shown in Eq. (2), the mechanical driving force in the ferroelectric film is related with h_{te} via the stress, which influences the domain structure evolution according to the time-dependent Ginzburg-Landau equation. Since the solving process is numerical, we cannot write down the analytical expressions.

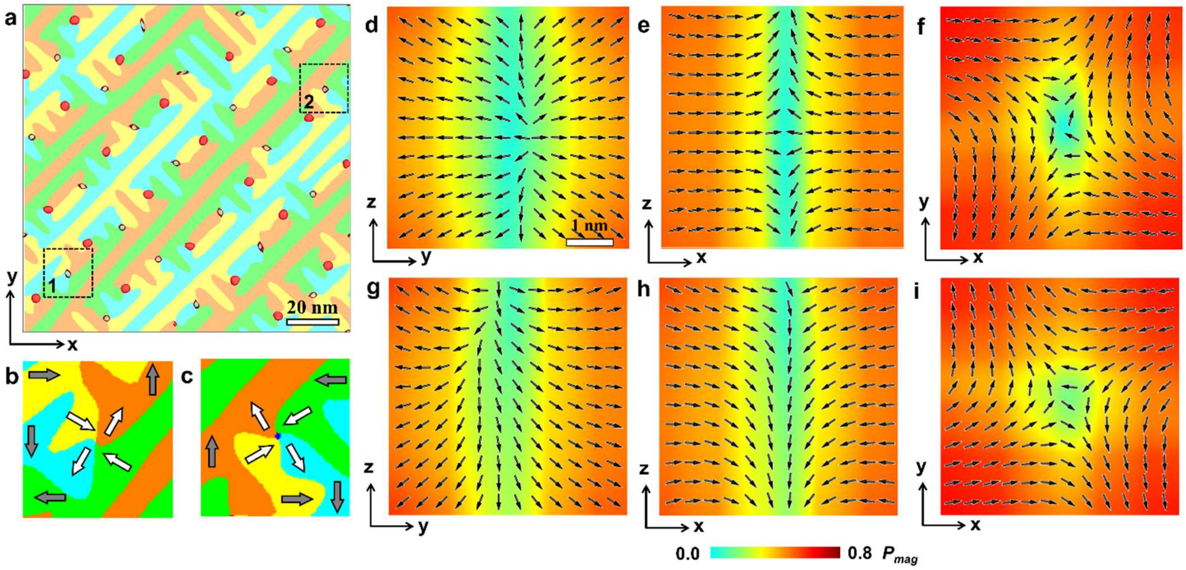

Supplementary Fig. 1│ The local polarization distribution of an abnormal BP-AM and one typical antimeron not associated with a Bloch point. (a) The horizontal slice of the PTO film overlaid with the isosurface of $P_{mag} = 0.55\ P_s$ where an abnormal BP-AM and one typical antivortex not associated with a Bloch point are marked by the dashed box 1 and 2, respectively. (b, c) The zoom-in slices of the two antimerons in (a). (d-f) The slices perpendicular to the *x* (d), *y* (e) and *z* (f) axes for the region "1" in (a). (g-i) The slices perpendicular to the *x* (g), *y* (h) and *z* (i) axes for the region "2" in (a). The normalized polarization vectors are overlaid therein.

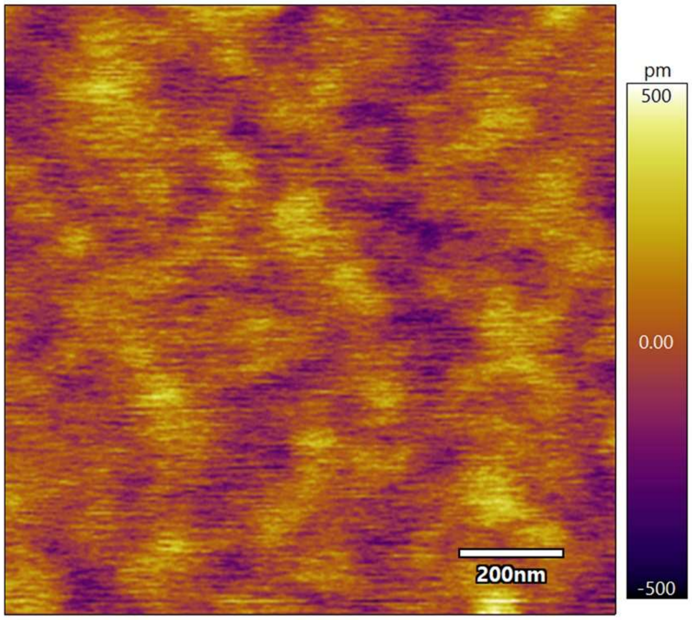

Supplementary Fig. 2 │The surface tomography acquired by atomic force microscope (AFM) showing a smooth surface of the trilayer PTO films.

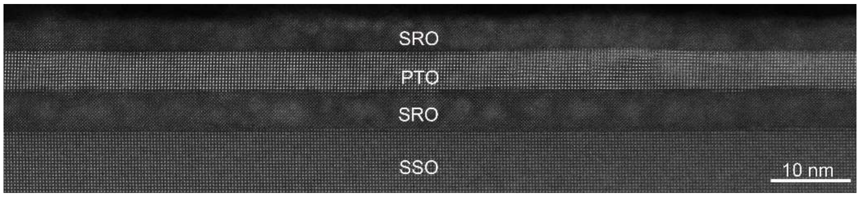

Supplementary Fig. 3 │The cross-sectional low-magnification HAADF-STEM image of the trilayer PTO films.

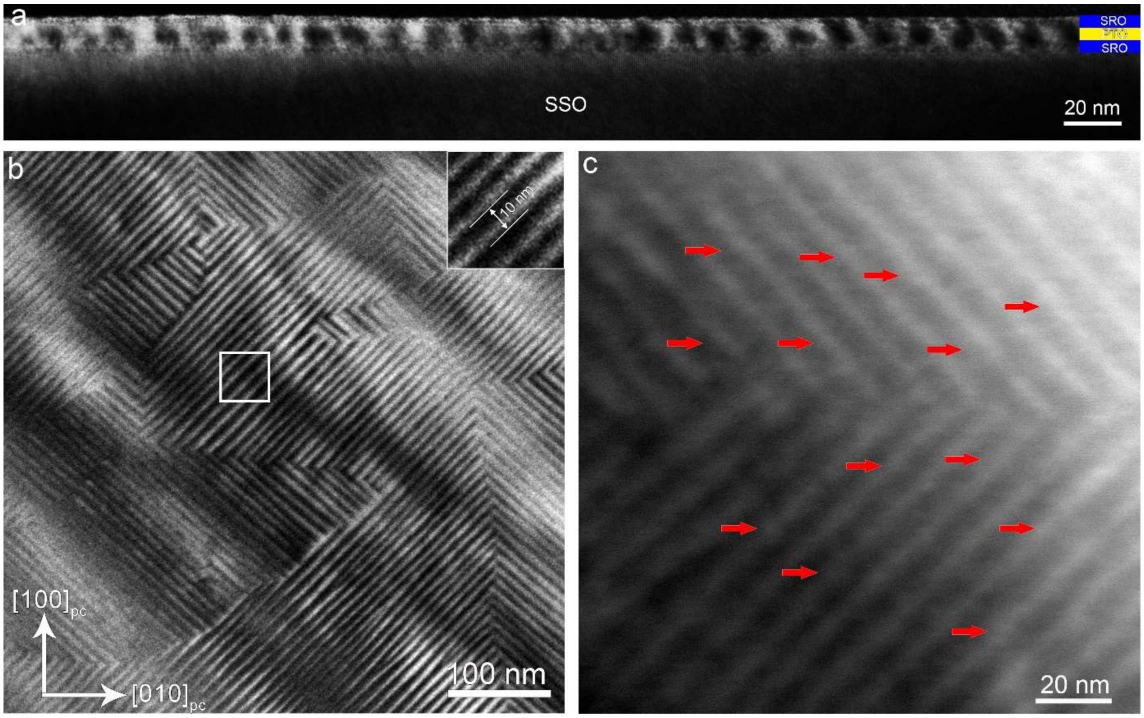

Supplementary Fig. 4 │Low-magnification TEM and STEM observations for the trilayer PTO films. (a) A low-magnification cross-sectional TEM image displays dot contrasts in the PTO layer. (b) A low-magnification planar-view dark-field TEM image exhibits the regular stripe a_1/a_2-like domains. The inset is the magnified image corresponding to the area marked by a white box in Fig. R2b, which indicates that the width of these stripe a_1/a_2-like domains is about 10 nm. (c) A planar-view HAADF-STEM image acquired by the defocus mode shows dot contrast fluctuations at stipe domain walls.

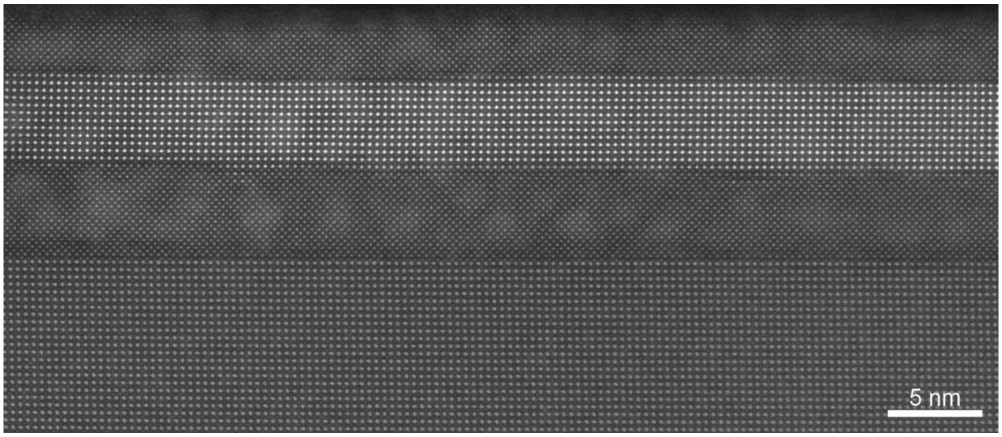

Supplementary Fig. 5 │The original high-resolution HAADF-STEM image for acquiring the polarization map of Fig. 3b.

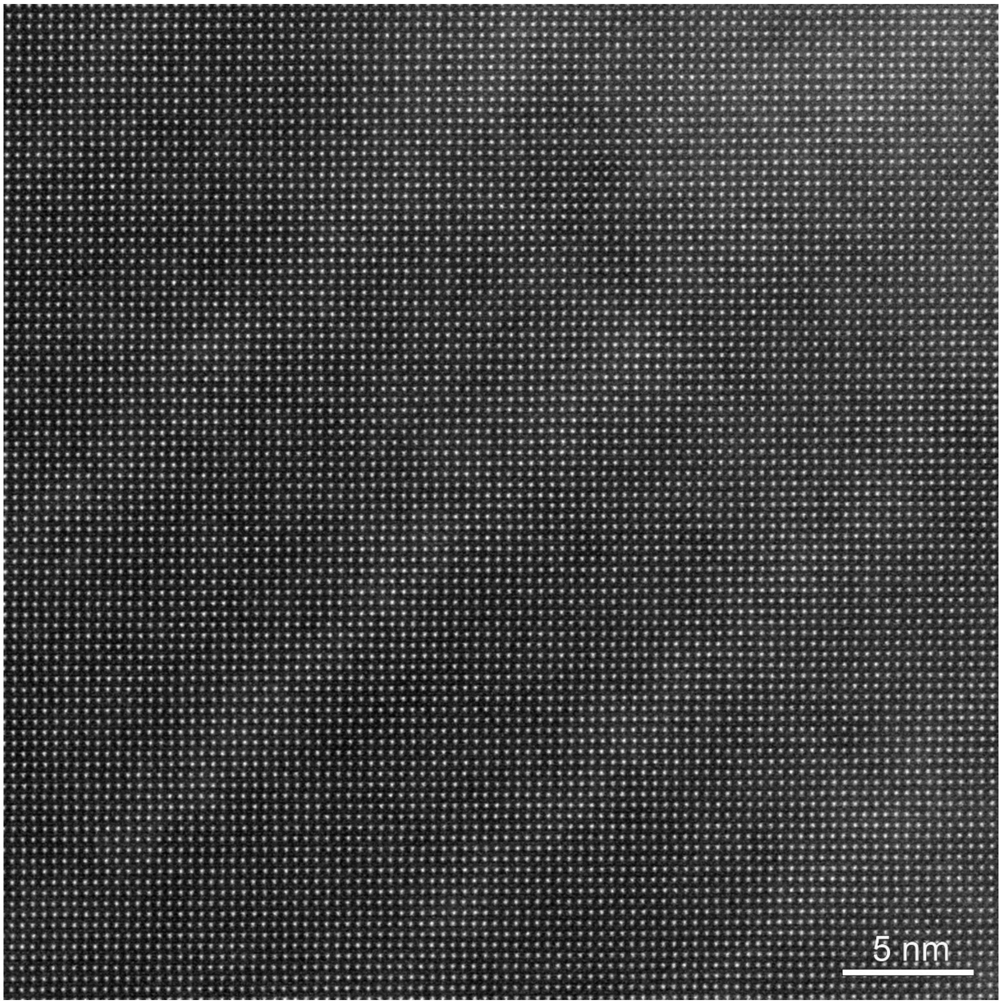

Supplementary Fig. 6 │The original high-resolution HAADF-STEM image for acquiring the polarization map of Fig. 3e.

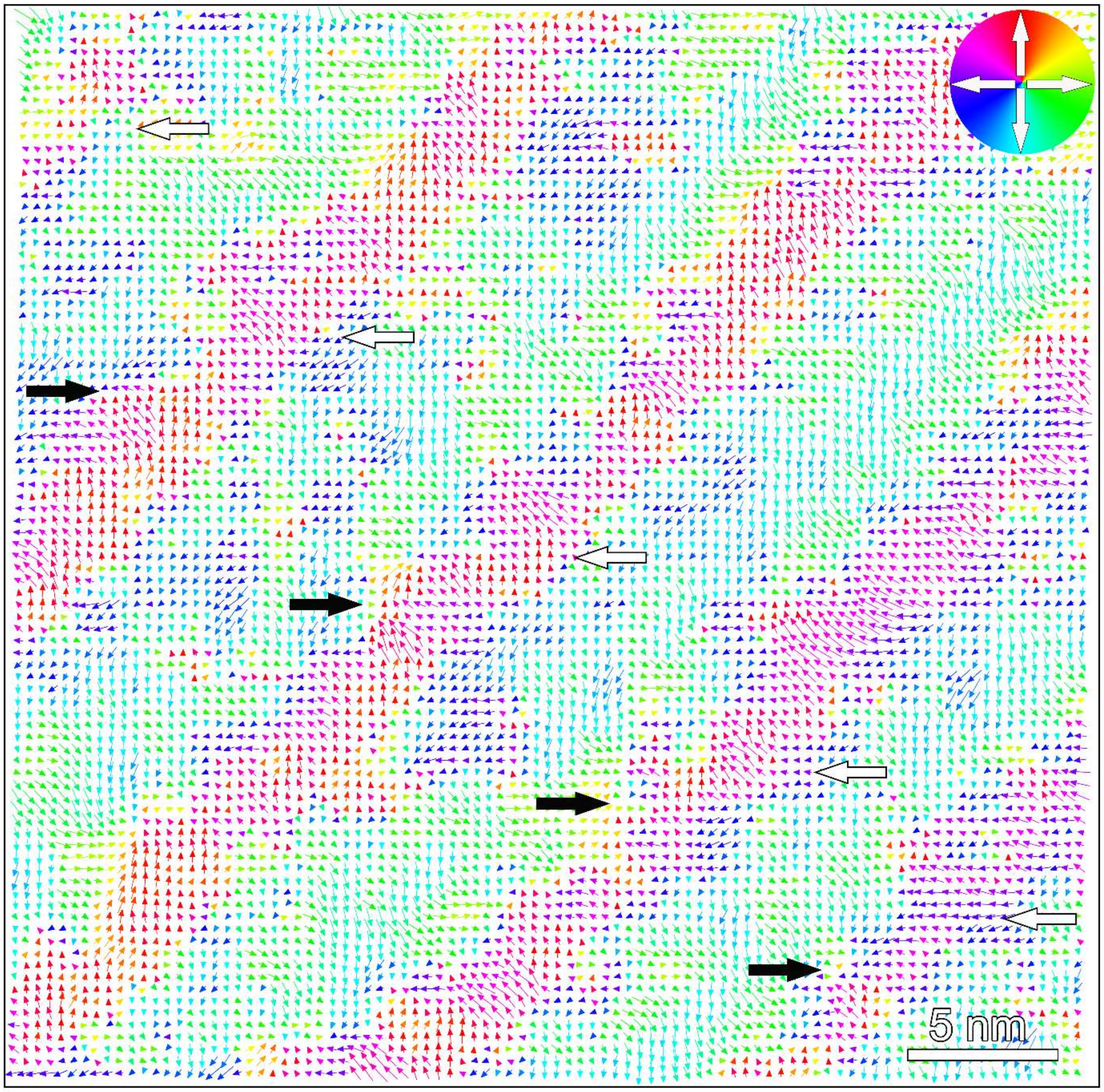

Supplementary Fig. 7 │The original $-\delta_{Ti}$ vector map based on atomic-resolved planar-view HAADF-STEM image. The black and white arrows point to the“head-to-head” and “tail-to-tail” domain walls, respectively.

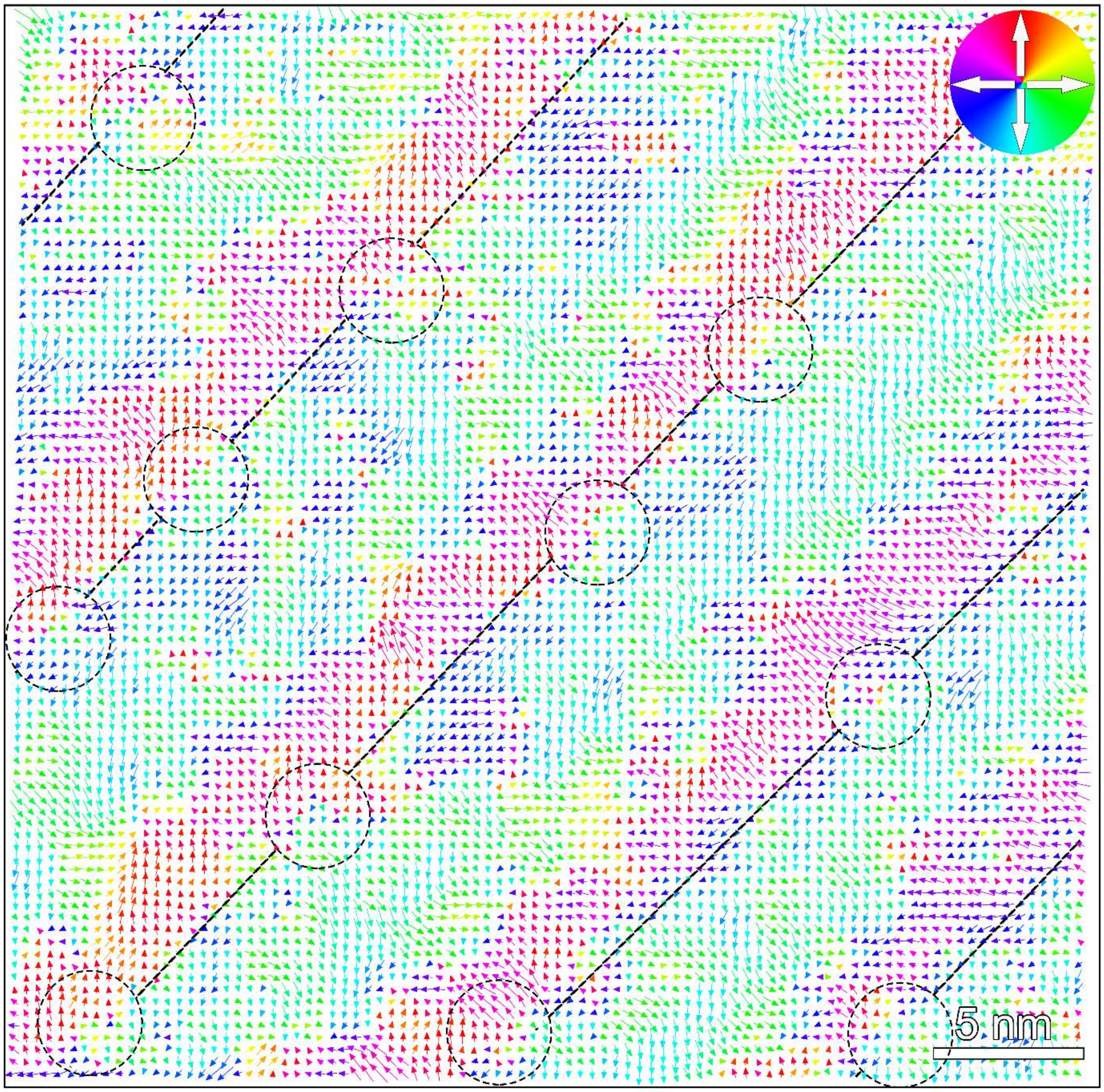

Supplementary Fig. 8 │The $-\delta_{Ti}$ vector map showing divergent polarization patterns (black dashed circles) at "tail-to-tail" domain walls (black dashed lines). The arrows with different colors denote the polarization directions of PTO unit cells.

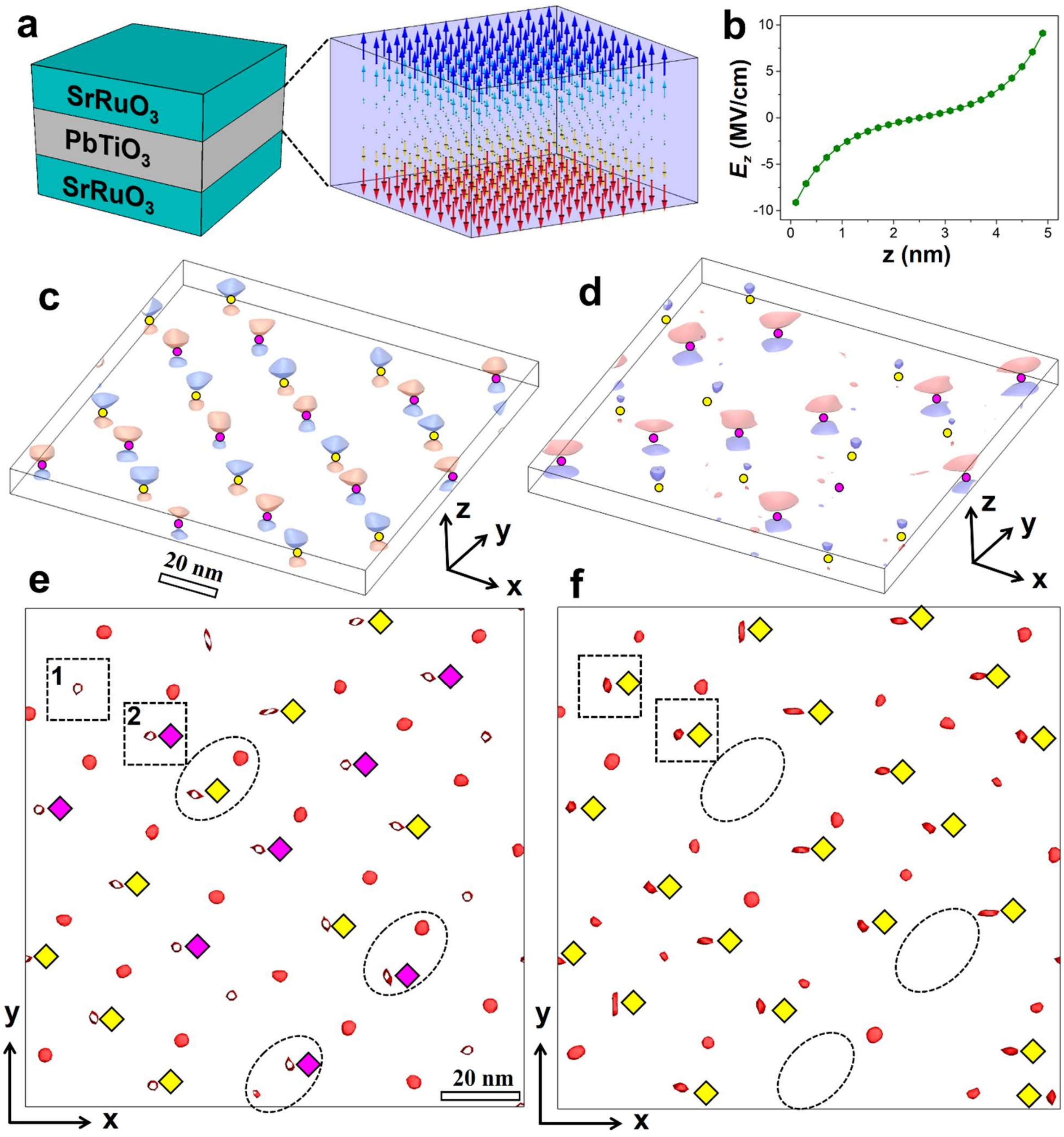

Supplementary Fig. 9 | The phase-field simulations of applying a convergent-type OP electric field. (a, b) The schematic distribution (a) and the line profile (b) of the electric field due to the work function difference between $SrRuO_3$ and $PbTiO_3$. The profile of the electric field is assumed to be $E_z = E_0\left[e^{-z/z_0} - e^{(z-h_f)/z_0}\right]$, where E_0 is the maximal electric field at the interface (9.1 MV/cm), h_f is the film thickness (5 nm) and z_0 is the characteristic length relating with the decay of the electric field (0.8 nm). (c) The isosurfaces of $P_z = \pm\, 0.55\ P_s$ without the electric field. (d) The isosurfaces of $P_z = \pm\, 0.70\ P_s$ with the application of the electric field. Brown and blue isosurfaces represent the positive and negative regions. (e, f) The isosurface of $P_{mag} = 0.55\ P_s$ (red) before (e) and after (f) the application of the electric field. CCD- and DDC-type Bloch points are marked by purple and yellow diamonds, respectively. Only BP-AM's are marked for a better visual effect. The detailed

variation of two representative antimerons are marked by two dashed boxes and shown in Supplementary Fig. 10. Three pairs of BP-M's and BP-AM's annihilate due to the effect of electric field and marked by dashed ellipses.

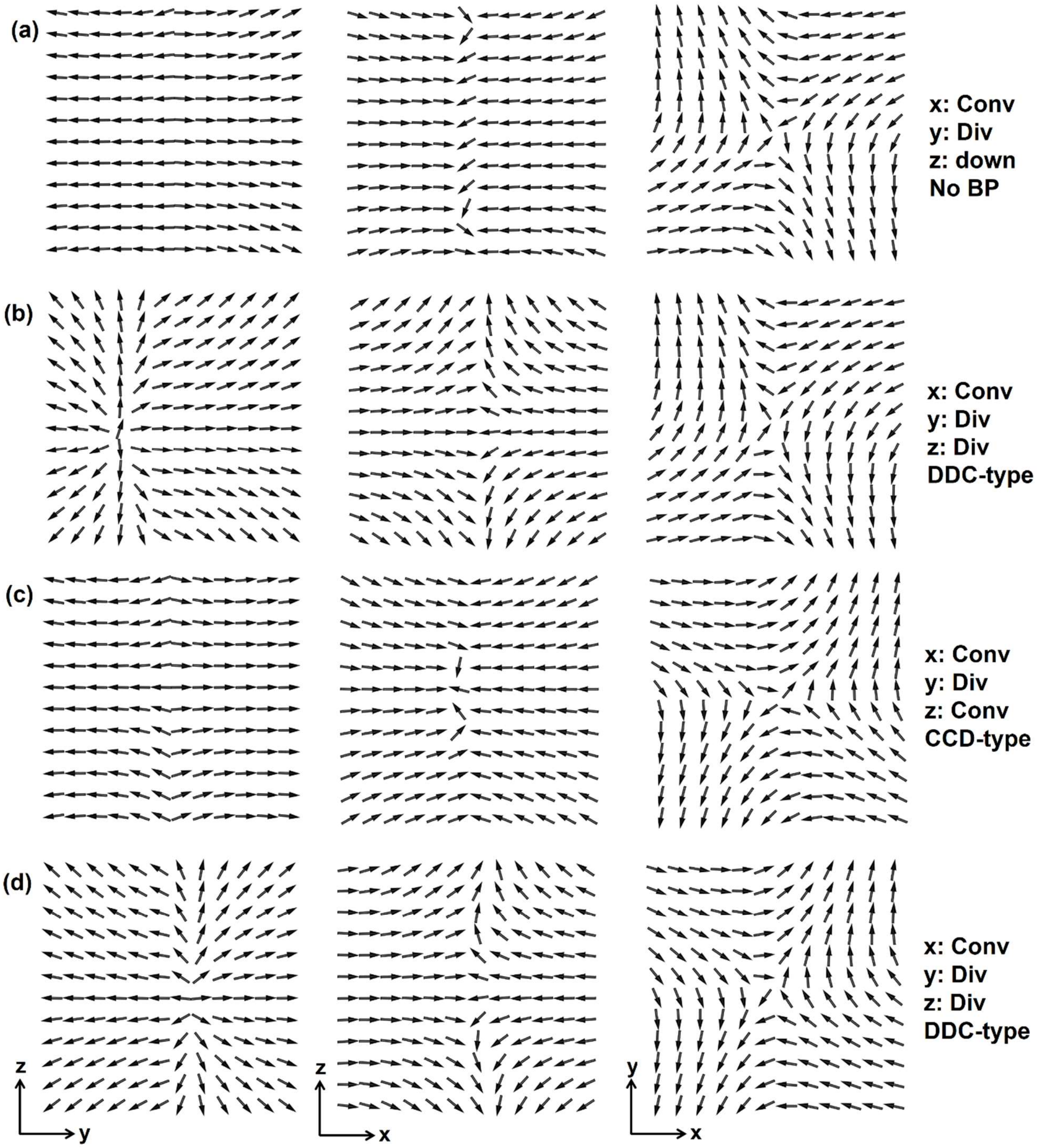

Supplementary Fig. 10 | The variation of two antimerons after the effect of electric field. (a, b) and (c, d) correspond to the dashed boxes "1" and "2" in Supplementary Fig. 9(e), respectively. (a) and (c) are the initial states and (b) and (d) are the state under the electric field. The left, middle and right columns are the slices perpendicular to the *x*, *y* and *z* axes, respectively.

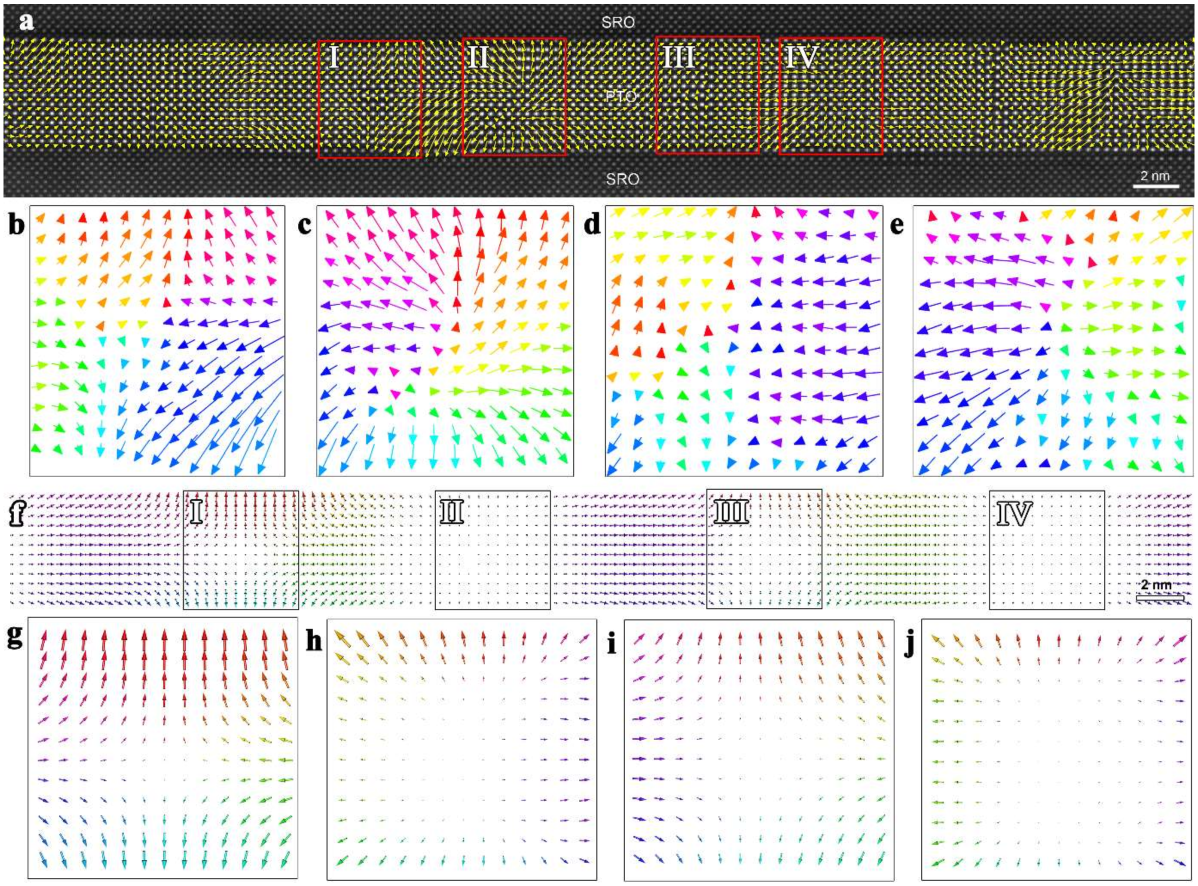

Supplementary Fig. 11 | The comparison of experimental and simulation cross-sectional polarization configurations. (a) Superposition of reversed Ti-displacement vectors ($-\delta_{Ti}$) and atomic-resolved HAADF-STEM image. The $-\delta_{Ti}$ vectors of PTO unit cells were shown as yellow arrows. (b-e) Magnified $-\delta_{Ti}$ vector maps showing four typical polarization patterns corresponding to the four red boxes in (a). (f) A cross-sectional slice of the simulated polarization distribution in Supplementary Fig. 9(d). (g-j) Magnified polarization vectors corresponding to the four black boxes in (f).

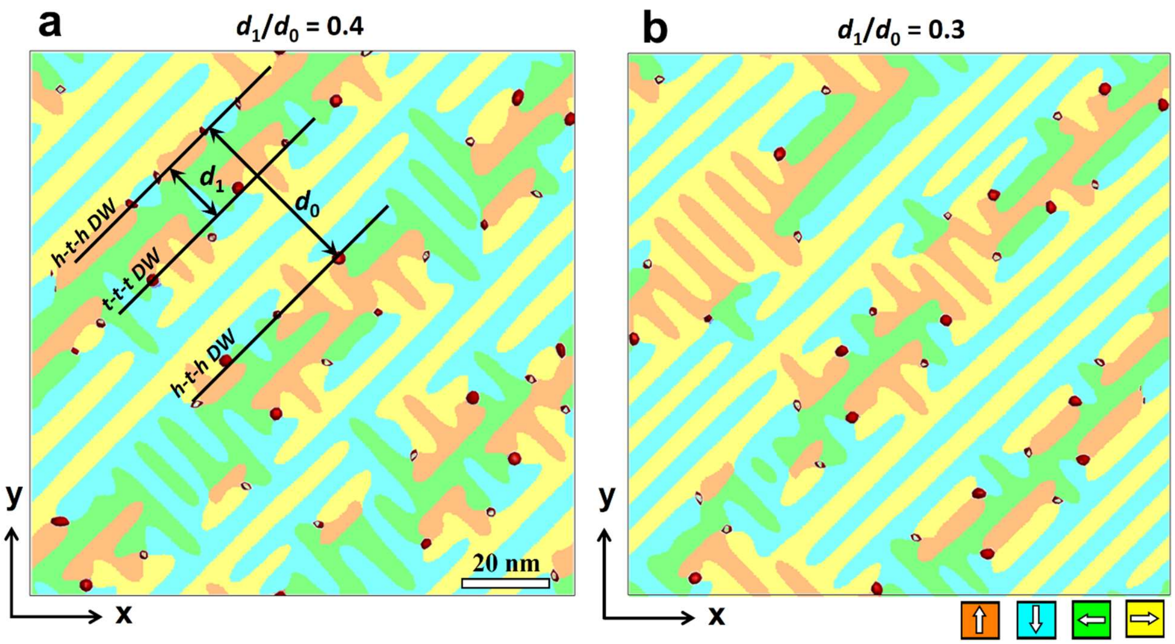

Supplementary Fig. 12 | The domain structures from the models with different initial distances between DWs. d_1 is the distance between the h-t-h and t-t-t DWs and d_0 is the distance between two h-t-h DWs. (a) $d_1/d_0 = 0.4$. (b) $d_1/d_0 = 0.3$. The results of $d_1/d_0 = 0.5$ is shown in Fig. 2(a). The number of Bloch points associated with merons decreases from 24 to 21 and 16 when the distance ratio d_1/d_0 decreases from 0.5 to 0.4 and 0.3.

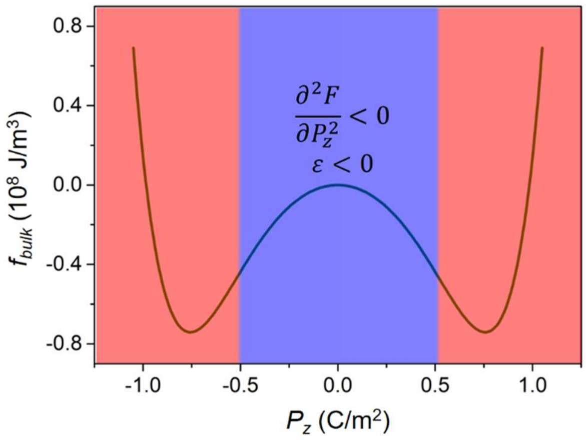

Supplementary Fig. 13 | The bulk free energy density as the function of polarization for ferroelectric $PbTiO_3$. The red and blue regions show the positive and negative capacitances.

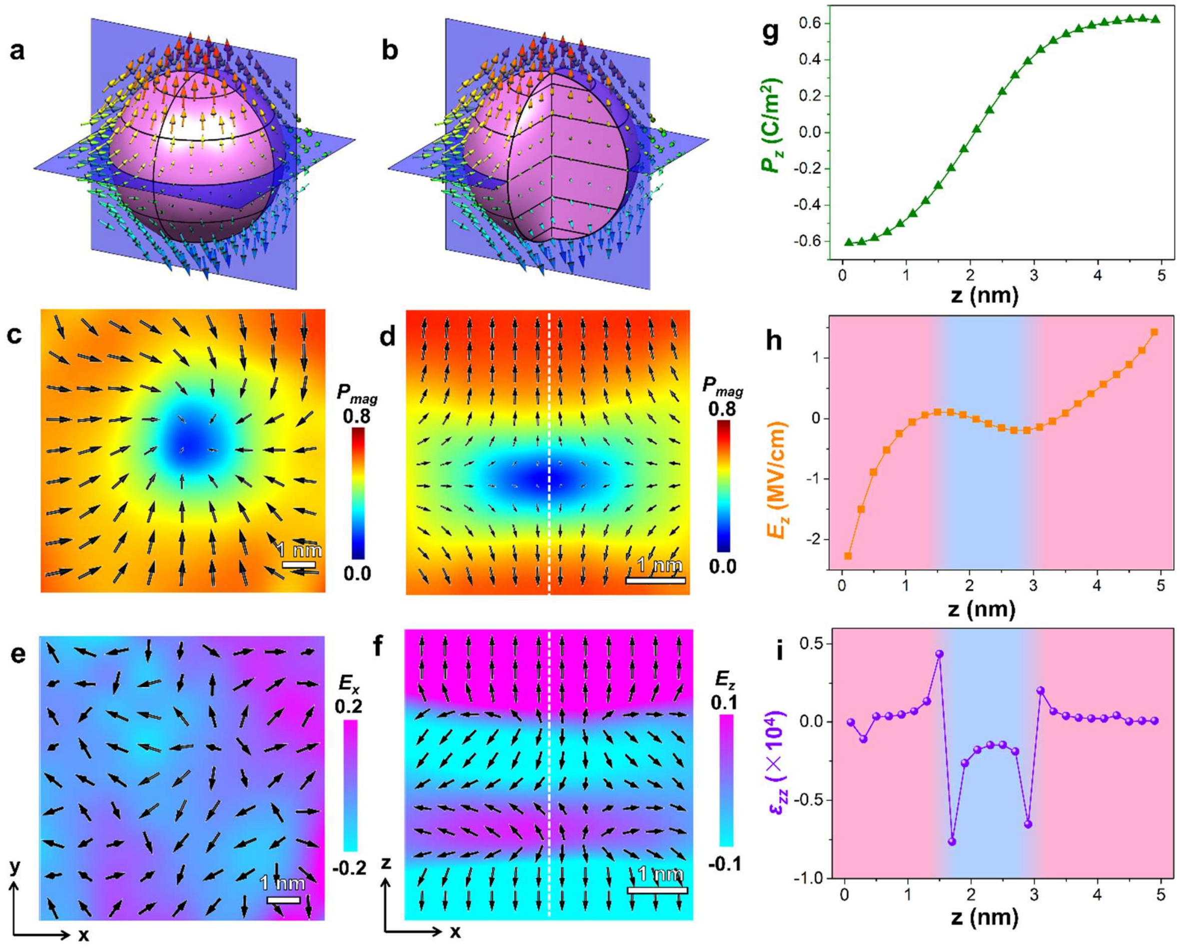

Supplementary Fig. 14 | Local negative capacitance around a CCD-type Bloch point predicted by the phase-field simulations. (a, b) The local polarization around a CCD-type Bloch point with the *xy* and *xz* cross-sections passing through the Bloch point. (c, d) The mappings of the polarization magnitude overlaid with the polarization vectors of the horizontal (c) and vertical (d) slices of the Bloch point in (a). (e, f) The corresponding mappings of the electric fields of the two slices in (c, d). (g-i) The profiles of the polarization (g), electric field (h) and ε_{zz} (i) along the dashed lines in (d) and (f). The red and blue background colors represent the regions of positive and negative capacitances, respectively.

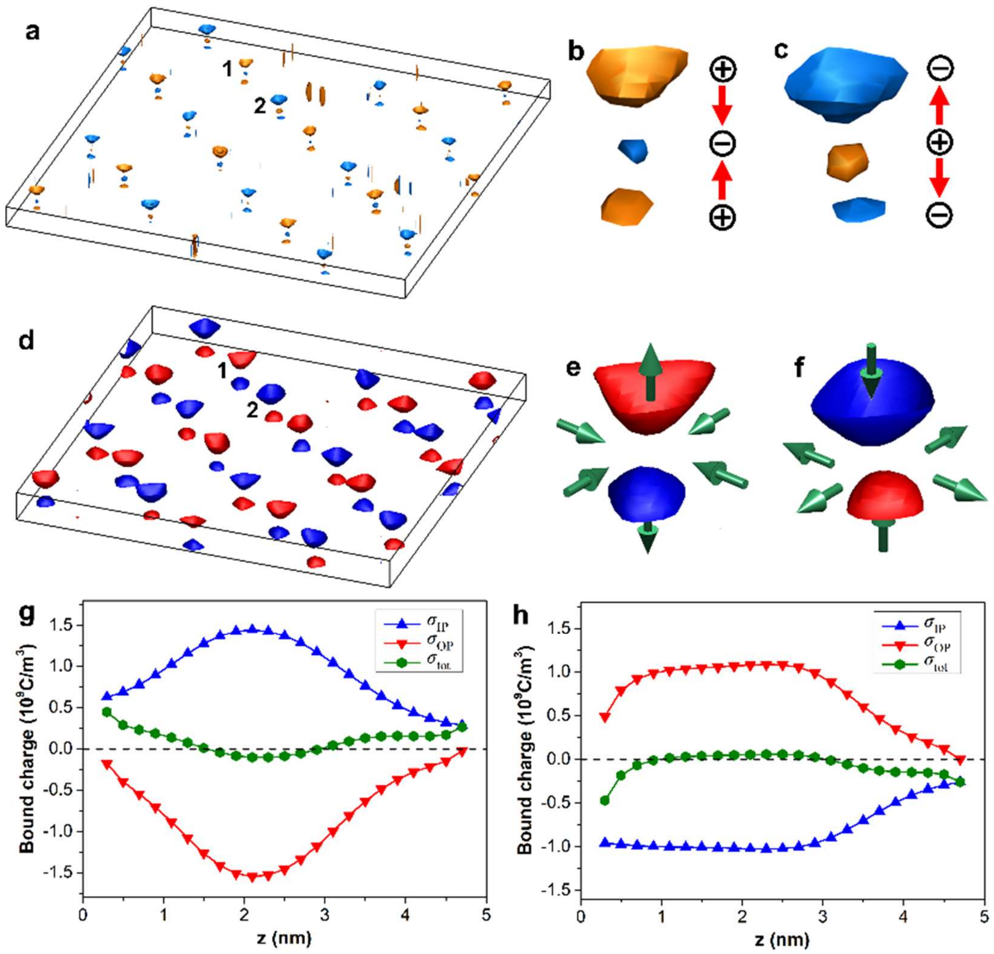

Supplementary Fig. 15 | The bound charge analysis for the two types of Bloch points. (a) The isosurfaces of $\sigma = +1{\times}10^8$ C/m^3 (brown) and $\sigma = -1{\times}10^8$ C/m^3 (light blue) of the PTO film containing the Bloch point lattice. To reveal more clearly the distribution of bound charge around the Bloch points, only the middle 3 nm out of the 5 nm film was presented. (b, c) The zoom-in images of the CCD- (b) and DDC-type (c) Bloch points marked by "1" (b) and "2" (c) in (a), respectively. (d) The isosurfaces of $P_z = +\ 0.5\ P_s$ (red) and $P_z = -\ 0.5\ P_s$ (blue). (e, f) The zoom-in images of the two regions marked by "1" (e) and "2" (f) in (d). (g, h) The profiles of in-plane (σ_{IP}), out-of-plane (σ_{OP}) and total (σ_{tot}) bound charges along the vertical lines through the two Bloch points marked by "1" (g) and "2" (h) in (a).

Supplementary Table 1. Material parameters of $PbTiO_3$

$\alpha_1 = -1.73\times10^8$ $C^{-2}\cdot m^2\cdot N$	$\alpha_{11} = -7.3\times10^7$ $C^{-4}\cdot m^6\cdot N$	$\alpha_{12} = 7.5\times10^8$ $C^{-4}\cdot m^6\cdot N$
$\alpha_{111} = 2.6\times10^8$ $C^{-6}\cdot m^{10}\cdot N$	$\alpha_{112} = 6.1\times10^8$ $C^{-6}\cdot m^{10}\cdot N$	$\alpha_{123} = -3.7\times10^9$ $C^{-6}\cdot m^{10}\cdot N$
$G_{11} = 1.04\times10^{-10}$ $C^{-2}\cdot m^4\cdot N$	$G_{12} = -1.04\times10^{-10}$ $C^{-2}\cdot m^4\cdot N$	$G_{44} = 1.04\times10^{-10}$ $C^{-2}\cdot m^4\cdot N$
$C_{11} = 1.75\times10^{11}$ $N\cdot m^{-2}$	$C_{12} = 7.94\times10^{10}$ $N\cdot m^{-2}$	$C_{44} = 1.11\times10^{11}$ $N\cdot m^{-2}$
$Q_{11} = 0.089$ $C^{-2}\cdot m^4$	$Q_{12} = -0.026$ $C^{-2}\cdot m^4$	$Q_{44} = 0.034$ $C^{-2}\cdot m^4$
$\alpha_0 = 1.73\times10^8$ $C^{-2}\cdot m^2\cdot N$	$G_{110} = 1.73\times10^{-10}$ $C^{-2}\cdot m^4\cdot N$	$P_0 = 0.76$ $C\cdot m^{-2}$
