# Supplementary material for: Polar Bloch points in strained ferroelectric films
Source: Nat Commun. 2024 May 10;15:3949. doi: 10.1038/s41467-024-48216-1 (PMC11087520; doi:10.1038/s41467-024-48216-1)
Supplement: Supplementary file 3 — Reporting Summary [file 41467_2024_48216_MOESM3_ESM.pdf]

nature research

Corresponding author(s): X. L. Ma

Lasing Reporting Summary

Nature Research wishes to improve the reproducibility of the work that we publish. This form is intended for publication with all accepted papers reporting claims of lasing and provides structure for consistency and transparency in reporting. Some list items might not apply to an individual manuscript, but all fields must be completed for clarity.

For further information on Nature Research policies, including our data availability policy, see Authors & Referees.

• Experimental design

Please check: are the following details reported in the manuscript?

1. Threshold

Plots of device output power versus pump power over a wide range of values indicating a clear threshold

- [ ] Yes
- [x] No

Our research primarily focus on the Polar Bloch points in strained ferroelectric films. The laser with fixed wavelength and energy serves only for PbTiO3 film preparation and parameters such as the threshold are not significant.

2. Linewidth narrowing

Plots of spectral power density for the emission at pump powers below, around, and above the lasing threshold, indicating a clear linewidth narrowing at threshold

- [ ] Yes
- [x] No

Our research primarily focus on the Polar Bloch points in strained ferroelectric films. The laser with fixed wavelength and energy serves only for PbTiO3 film preparation and parameters such as linewidth narrowing are not significant.

Resolution of the spectrometer used to make spectral measurements

- [ ] Yes
- [x] No

Our research primarily focus on the Polar Bloch points in strained ferroelectric films. The laser with fixed wavelength and energy serves only for PbTiO3 film preparation and not for spectral measurements.

3. Coherent emission

Measurements of the coherence and/or polarization of the emission

- [ ] Yes
- [x] No

Our research primarily focus on the Polar Bloch points in strained ferroelectric films. The laser with fixed wavelength and energy serves only for PbTiO3 film preparation and the coherence and/or polarization of the emission are not significant.

4. Beam spatial profile

Image and/or measurement of the spatial shape and profile of the emission, showing a well-defined beam above threshold

- [ ] Yes
- [x] No

Our research primarily focus on the Polar Bloch points in strained ferroelectric films. The laser with fixed wavelength and energy serves only for PbTiO3 film preparation and parameters such as beam spatial profile are not significant.

5. Operating conditions

Description of the laser and pumping conditions
Continuous-wave, pulsed, temperature of operation

- [x] Yes
- [ ] No

We have provided detailed descriptions of the operating conditions in the Methods section, as"pulsed laser deposition (PLD), using the Coherent ComPex PRO 201 F KrF excimer laser (y= 248 nm)".

Threshold values provided as density values (e.g. W cm^{-2} or J cm^{-2}) taking into account the area of the device

- [ ] Yes
- [x] No

We did not specify the threshold as a density value as this is not relevant to our study. But we have added the energy density of the laser fluence when films deposition in the Methods part, as"During SRO layers deposition, an oxygen pressure of 7 Pa, laser energy of 1.7 J cm-2 and repetition rate of 4 Hz were used, while an oxygen pressure of 10 Pa, laser energy of 2 J cm-2 and repetition rate of 4 Hz were used when depositing PTO layer."

6. Alternative explanations

Reasoning as to why alternative explanations have been ruled out as responsible for the emission characteristics
e.g. amplified spontaneous, directional scattering; modification of fluorescence spectrum by the cavity

- [ ] Yes
- [x] No

Our research primarily focus on the Polar Bloch points in strained ferroelectric films. The laser with fixed wavelength energy serves only for film preparation and parameters such as amplified spontaneous and directional scattering are not significant.

7. Theoretical analysis

Theoretical analysis that ensures that the experimental values measured are realistic and reasonable
e.g. laser threshold, linewidth, cavity gain-loss, efficiency

- [ ] Yes
- [x] No

Our research primarily focus on the Polar Bloch points in strained ferroelectric films. The laser with fixed wavelength energy serves only for film preparation and parameters such as laser threshold, linewidth, cavity gain-loss, efficiency are not significant.

8. Statistics

Number of devices fabricated and tested	☒ Yes<br>☐ No	We have fabricated one sample of SRO/PTO/SRO grown on SSO substrate with sizes of 2.5 mm*10 mm. Five tests were performed to acquire the domains and polarization distribution in this sample by transmission electron microscope and an aberration-corrected scanning transmission electron microscope (Fig. 3, Supplementary Fig. 4).One test was performed to acquire the surface tomography by atomic force microscope.
Statistical analysis of the device performance and lifetime (time to failure)	☐ Yes<br>☒ No	Our research primarily focus on the Polar Bloch points in strained ferroelectric films. We just deposited SRO/PTO/SRO trilayer as the prototype system to modulate the polar topological domains. There was no statistical analysis due to no specific device in this study.
